# Antigen-specific CD8+ memory stem T cells generated from human peripheral blood effectively eradicate allogeneic targets in mice

**DOI:** 10.1186/s13287-018-1080-1

**Published:** 2018-12-07

**Authors:** Liping Guan, Xiaoyi Li, Jiali Wei, Zhihui Liang, Jing Yang, Xiufang Weng, Xiongwen Wu

**Affiliations:** Department of Immunology, School of Basic Medicine, Tongji Medical College, Huazhong University of Science and Technology, 13 Hangkong Rd, Wuhan, 430030 China

**Keywords:** T memory stem cells, Allogeneic antigen specificity, Preparation in vitro, Adoptive immunotherapy

## Abstract

**Background:**

As the implantation and long-term existence of tumor-specific T cells in host are the prerequisite for adoptive immunotherapy, memory stem T cells (T_SCM_) with self-renewal and differentiation capacity show the greatest potential to implant and long-term exhibit function in vivo, compared with other T cells of differentiation stages. Hence, tumor-specific T_SCM_ have become potential candidate for adoptive T cell therapy of cancer. Here, we reported a protocol to generate allogeneic antigen-specific CD8+ T_SCM_ cells from human PBLs.

**Methods:**

To prepare allogeneic antigen-specific CD8+ T_SCM_, we used an LCL named E007 of defined HLA allotyping as simulator, a co-culture of E007 and allogeneic PBLs was carried out in the presence of differentiation inhibitor TWS119 for 7 days. Sorting of proliferation cells ensured the E007-specificity of the prepared T_SCM_ cells. The sorted lymphocytes underwent further expansion by cytokines IL-7 and IL-15 for further 7 days, making the E007-specific CD8 + T_SCM_ expanded in number. The stem cell and T memory cell properties of the prepared CD8+ T_SCM_ were observed in NOD-SCID mice.

**Results:**

Our protocol began with 1 × 10^7^ PBLs and resulted in 2 × 10^7^ E007-specific CD8+ T_SCM_ cells in 2 weeks of preparation. The prepared T_SCM_ cells exhibited a proliferative history and rapid differentiation into effector cells upon the E007 re-stimulation. Importantly, the prepared T_SCM_ cells were able to exist long and reconstitute other T cell subsets in vivo, eradicating the E007 cells effectively after transferred into the LCL burden mice.

**Conclusions:**

This protocol was able to prepare allogeneic antigen-specific CD8+ T_SCM_ cells from human PBLs. The prepared T_SCM_ showed the properties of stem cells and T memory cells. This study provided a reference method for generation of antigen-specific T_SCM_ for T cell adoptive immunotherapy.

**Electronic supplementary material:**

The online version of this article (10.1186/s13287-018-1080-1) contains supplementary material, which is available to authorized users.

## Background

Adaptive immunity is an effective strategy for cancer treatment. T cells are the main force to combat microbes and cancer cells in adaptive cells [1, 2]. However, T cell is heterogeneous, which exists in a continuum of differentiation states. According to differentiation stages, T cells can be divided into naive T cells (T_N_), memory stem cells (T_SCM_), central memory cells (T_CM_), effector memory cells (T_EM_) and differentiated effectors (T_EF_) [3, 4]. Adoptive T cell therapy (ACT) for clinical application is normally based on the use of terminally differentiated T_EF_ cells, which have short lifespan and inferior engraftment capacities [5–7]. T_SCM_ is defined as the earliest developmental stage of memory T cells, which possesses capacities of self-renewal and differentiation [5, 8]. Phenotypically, T_SCM_ can be identified by the expression of CD3+ CD45RA+ CD62L+ CD95+ CCR7+ CD28+ [5, 9–11]. T_SCM_ has longer lifespan, as it is reported T_SCM_ is able to be tracked in vivo for 12 years after infusion of genetically modified lymphocytes [7]. In particular, T_SCM_ mediates longer and more robust efficiency of tumor rejection in vivo compared to other memory and effector subsets [5, 6, 8, 12–14]. It is these characteristics that make T_SCM_ a good candidate for immunological cytotherapy of cancer.

T_SCM_ constitutes a small proportion of the T cell subset, 2–4% of the total T cells in peripheral blood [5, 8]. The premise of antigen-specific T_SCM_ used for clinical therapy is to accomplish in vitro T_SCM_ preparation in a large number. It is demonstrated that small molecule chemical inhibitor TWS119, a potent inhibitor of the serine–threonine glycogen synthase kinase 3β (GSK-3β) able to induce the Wnt-β-catenin signaling, helps the enrichment of T_SCM_ through differentiation inhibition both in mice and in human [15–17]. On the other hand, T_SCM_ can be amplified with cytokines IL-7 and IL-15; IL-21 is also reported to promote the generation of T_SCM_ under ex vivo culture conditions [6, 18–26].

Allogeneic hematopoietic stem cell transplantation (allo-SCT) is an effective immunotherapeutic approach with curative potential in patients with malignancies. The therapeutic basis is mainly dependent on the donor T cell alloresponses against the recipient’s malignant cells named as graft versus leukemia (GVL) or graft versus tumor (GVT) effect [27]. T cell responses to alloantigen are of peptide-MHC complex (pMHC) specificity in the same way as those to nominal antigen [28, 29]. Transfer of alloreactive T cells with defined specificity, such as for leukemia- or tumor-associated antigens, is proposed to separate the GVL or GVT effect from the deleterious graft versus host disease (GVHD) [30, 31]. As the implantation and long-term existence of tumor-specific T cells in host are the prerequisite for adoptive immunotherapy, it is of importance to prepare alloantigen-specific T_SCM_ cells in vitro.

In this study, we explored a methodology of alloantigen-specific T_SCM_ (allo-specific T_SCM_) preparing in vitro. Although both CD4+ and CD8+ T_SCM_ cells are reported, we focused on CD8+ T_SCM_ cells. The T_SCM_ cells were generated in an allogeneic co-culture, enriched by TWS119, then sorted with proliferation, and finally expanded by IL-7 and IL-15. Our protocol prepared 2 × 10^7^ allo-specific T_SCM_ cells from 1 × 10^7^ PBLs. An LCL burden mouse model was introduced in for the T_SCM_ cell behavior in vivo. LCL cells were human B lymphoblastoid cells immortalized by EB virus infection. Although this model would reflect lymphoproliferative disorders, the LCL cells in mice could act as allogeneic targets to measure eradication efficacy of T cells in vivo in our study. Importantly, the prepared T_SCM_ cells exhibited both stem cell and memory T cell properties, and were able to implant and effectively eradicate the targets after adoptive transfer into mice. This study provided a practical method for generation of allo-specific T_SCM_ cells in vitro, which would be expected to be referred in preparation of allogeneic T_SCM_ grafts with defined antigen specificity for the purpose of adoptive immunotherapy.

## Materials and methods

### Peripheral blood lymphocyte isolation

Peripheral blood was obtained from healthy donors after informed consent under a protocol approved by the Ethics Committee of Tongji Medical College, Wuhan, China. Peripheral blood mononuclear cells (PBMCs) were isolated by centrifugation through a ficoll-hypaque gradient (Ficoll-Hypaquedensity 1.077 g/ml) and cultured in RPMI-1640 medium supplemented with 10% fetal bovine serum (FBS), then placed in dish for 2 h to remove the adherent cells. The non-adherent cells were collected as peripheral blood lymphocytes (PBLs) for the co-culture.

### Cell lines and antibodies

The EB virus (EBV) transformed B lymphoblastoid cell lines (LCLs) E007 and E001 were established in our lab according to reported protocol [32]. HLA typing for LCLs was performed with PCR-SSP [33]. The HLA class I alleles of E007 and E001 are mismatched, E007 carried A*110x, 310x; B*510x, 550x; C*030x, 150x; and E001 A*020x, 240x; B*460x, 540x; C*010x, 080x.

Fluorescent antibodies for cells staining included human CD3-APC-Cy7 (clone HIT3a), CD8-BV510 (clone SK1), CD3-Percp-Cy5.5 (clone HIT3a), CD62L-PE-Cy7 (clone DREG-56), CD45RA-APC (clone HIT100), CD45RA-BV421 (clone HI100), CD95-PE (clone DX2), CXCR7-PE (clone 10D1-J16), CD28-APC (clone CD28.2), IL-2-APC (clone MQ1-17H12), IFN-γ-PE (clone 4S.B3), TNF-α-PE (clone MAb11), the above antibodies were purchased from BioLegend, USA. Corresponding isotype for each antibody were used as isotype control. Cells were analyzed on a BD LSR flow cytometer. T cell subsets were determined using fluorescence minus one (FMO) controls for interesting antibody.

### Allogeneic co-culture and allo-specific T_SCM_ preparation

Allogeneic PBLs were co-cultured with the E007 for raising alloreactive T_SCM_ cells. PBLs were stained with 5 μΜ carboxyfluorescein diacetate succinimidyl ester (CFSE, sigma) for 8 min at 37 °C and washed with RPMI1640 supplemented with 10% FBS three times. E007 were inactivated by irradiating (2.0 Gy). PBLs were mixed with E007 on day 0 at a ratio of 5:1 with 5 μM TWS119 in the co-culture for the first week. PBLs cultured alone used as control. TWS119 and medium were replaced on day 4, and cells were counted by Trypan blue dye exclusion. As the allo-specific T cells showed proliferation in the co-culture, CFSEdim cells were sorted by FACS with BD FACS AriaII on day 7. Sorted cells were expanded by cytokines IL-7 and IL-15 (Peprotech, USA) of 25 ng/ml each for a further week.

T cell subsets in the co-culture bulks were identified by their surface markers, CD3+ CD8+ CD45RA+ CD62L+ CCR7+ CD95+ CD28+ for T_SCM_, CD3+ CD8+ CD45 RA- CD62L+ for T_CM_, CD3+ CD8+ CD45RA- CD62L- for T_EM_, and CD3+ CD8+ CD45 RA+ CD62L- for T_EF_.

### Preparation of allo-specific T_EM_ and T_EF_ for mouse transfer

Allo-specific T_EM_ and T_EF_ cells were prepared by a co-culture of allogeneic PBLs with E007 set up as above, but no TWS119 was added. CFSEdim cells were sorted on day 7. Sorted cells were further expanded by 300 U/ml IL-2 (Peprotech, USA) instead of IL-7 and IL-15 for a further week.

### In vitro T_SCM_ cells differentiation assay and intracellular cytokine staining

The prepared T_SCM_ cells were incubated with E007, E001 cells and Dynabeads Human T-Activator CD3/CD28 (α-CD3/CD28) (Gibco, USA), respectively, in a 5:1 ratio. Cells were collected at 6, 12, and 24 h, and labeled with corresponding fluorescent antibodies for differentiation assay. The T_SCM_ cells incubated with the above stimulations in the presence of 1 × BFA (eBioscience, USA) were collected for intracellular IFN-γ, IL-2, and TNF-α staining after 4 h incubation. Samples were analyzed on BD Verse flow cytometer. The data analysis was performed with Flowjo software version 10.0 (Tree Star).

### Quantitative real-time PCR

T cell subsets in the co-culture were sorted by BD Aria II flow cytometer based on the corresponding phenotypes on day 7. Total DNA of the T cells was isolated using a genomic Extraction kit (Takara, Japan), following manufacturer’s instructions. TRECs of T cell subsets were detected using fluorescently quantitative PCR kit (Takara, Japan) with TREC-specific primers [34] on Bio-Rad CFX Sequence detection system.

### Animal experiments

6-week-old female NOD-SCID mice were purchased from Vital River Lab Animal Co, Ltd. (Beijing; a distributor of the Jackson Laboratory). Animal experiments in this study were approved by the Ethical Committee of Tongji Medical College. The mice were irradiated with 2.0 Gy and randomized in five groups (*n* = 5), then intravenous injection with 8 × 10^6^ E007 or E001 on day − 3. The mice were injected with the 1 × 10^7^ allo-specific T_SCM_ or allo-specific T_EM_ and T_EF_ on day 0. Blood samples were taken every week from mice after the T cell transfer. The number and phenotype of human T cells in mouse peripheral blood were determined by flow cytometry. The signs of GVHD were monitored daily. Mice were euthanized on day 35 and bone marrow and spleen were removed to detect human T cells and residual LCL cells. The LCL cells were detected by the latent membrane protein 1 (LMP1) of EB virus using antibodies LMP1-FITC (primary antibody: mouse anti-EBV LMP1, clone CS1–4, Abcom; secondary antibody: goat anti-mouse IgG-FITC, clone poly 4053, BioLegend) and flow cytometry.

### Immunofluorescence analysis for mouse spleen

Formaldehyde-fixed spleen specimens were embedded in paraffin and cut into sections for immunofluorescent detection of human CD3+ T cells with antibodies CD3-488 (primary antibody: rabbit anti-human CD3, clone SP7, ThermoFisher; secondary antibody: goat anti-rabbit lgG H&L-Alexa Fluor 488, Abcom) and LMP1-CY3 (secondary antibody: goat anti-mouse lgG H&L-Cy3, Abcom) respectively. Nuclei were stained with DAPI (BioLegend).

### Statistical analysis

The statistical significance of differences between two groups was assessed with a 2-tailed paired or unpaired *t* test. Comparisons of more than two groups were performed by one-way ANOVA with multiple comparison tests. Data are shown as the mean ± standard deviation (SD). Difference were marked as NS, *P* > 0.05; *P < 0.05; **P < 0.01, and ***P < 0.001. All the data obtained from the study was analyzed using SPSS 22.0 (IBM, USA).

## Results

### Our in vitro protocol is able to prepare allo-specific CD8+ T_SCM_ cells effectively

**Fig. 1:**
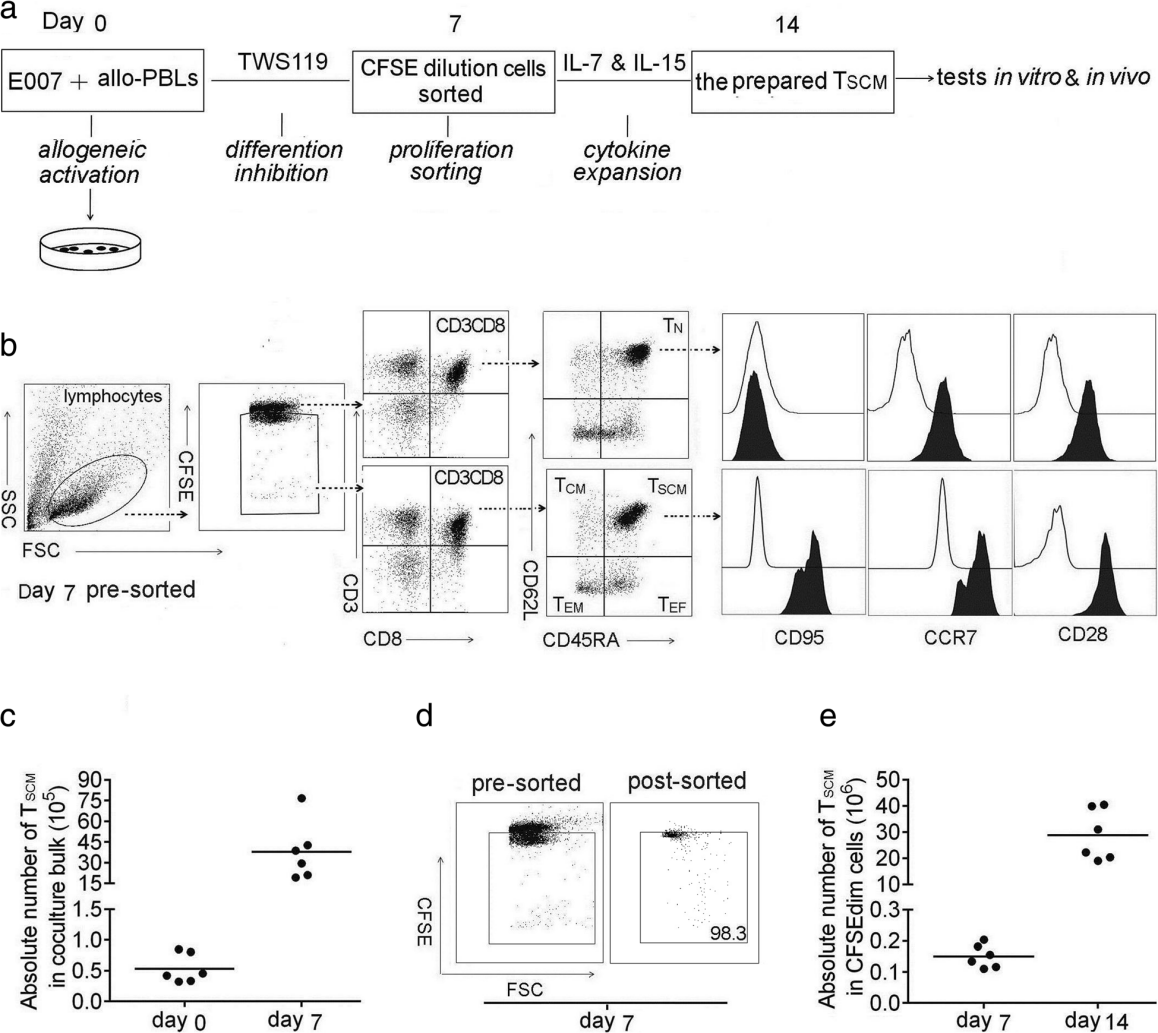
Allo-specific T_SCM_ cells are effectively prepared in vitro. **a** Protocol for preparation of allo-specific T_SCM_. The method began with setting up an allo-reactive co-culture by mixing of 2 × 10^6^ E007 cells and 1 × 10^7^ allogeneic PBLs (allo-PBLs) on day 0 (allogeneic activation), to generate allo-specific T cells. The presence of 5 μM TWS119 enriched T_SCM_ in the co-culture (differentiation inhibition). The CFSE diluted cells in the co-culture bulks were sorted by FACS on day 7 (proliferation sorting). The sorted cells were cultured with IL-7 and IL-15 (25 ng/ml each) to expand the T_SCM_ for the next 7 days (cytokine expansion). Then, the cultural bulks were used as the prepared T_SCM_ cells for the tests in vitro and in vivo included in this study. **b** Gating strategy for identification of CD8+ T_SCM_ subset in the co-culture bulks. Lymphocytes were identified based on SSC versus FSC, and proliferating cells were on a reduced fluorescence intensity of CFSE (CFSEdim). In the gated CFSEdim population, cells expressing CD3 and CD8 were selected for further CD45RA and CD62L analysis. CD8+ T_SCM_ showed the phenotype of CFSEdim CD3+ CD8+ CD45RA+ CD62L+, T_CM_ of CD45RA- CD62L+, T_EM_ of CD45RA- CD62L-, T_EF_ of CD45RA+ CD62L-, and CD95 was further detected to distinguish T_SCM_ from T_N_, the latter showed the phenotype of CFSE high CD95- in the co-culture bulks, but T_SCM_ of CFSEdim CD95+. The illustration was a representative of co-culture bulks analyzed by FCM on day 7 before sorting, the white peaks in CD95, CCR7, and CD28 plots were isotype controls. The broken arrows indicated the sequential gating strategy. **c** T_SCM_ numbers increased by 100 folds in the co-culture bulks with TWS119 enrichment for 7 days. Data are represented as mean ± SD of six individual experiments. **d** E007-specific T cells underwent proliferation in the co-culture, the CFSEdim cells were sorted by FACS with purity above 98%. **e** The sorted cells were further expanded by IL-7 and IL-15 for the next 7 days, the allo-specific T_SCM_ increased by another 150 folds. Data are represented as mean ± SD of six individual experiments

To prepare allo-specific T_SCM_, this study began with a co-culture of a simulator cells and allogeneic PBLs on day 0. The stimulator was an LCL named as E007 with defined HLA allotyping. Due to the difference in HLA alleles among random donors, allo-specific T_SCM_ cells were generated from T_N_ through proliferation during the co-culturing (Fig. 1a). The T_SCM_ were enriched in the presence of differentiation inhibitor TWS119, of which the optimal concentration was 5 μM in the allogeneic co-culture (Additional file 1: Figure S1A B). On day 7, the T_SCM_ cells in the co-culture bulks were defined by the phenotype CD3+ CD8+ CD45RA+ CD62L+ CD95+ CCR7+ CD28+ (Fig. 1b). The inhibition of differentiation in the allogeneic co-culture enriched the T_SCM_ numbers by 100 folds on day 7 (Fig. 1c). As the allo-specific T cells showed proliferation in the co-culture, sorting of proliferation cells ensured the antigen-specificity of the prepared T_SCM_ cells. The sorting reached above 98% purity of the proliferative cells (Fig. 1d). It would be rational that the prepared T_SCM_ cells were E007 specific. After sorting, the cells were cultured in the presence of IL-7 and IL-15 (25 ng/ml each) for the next 7 days. The allo-specific T_SCM_ increased by another 150 folds on day 14 (Fig. 1e). The lymphocyte distribution at this time not only showed the cultural bulks were mainly CD8+ T_SCM_ (60.1 ± 11.2%), but also contained a few CD4+ T_SCM_ (10.4 ± 8.16%), CD3- cells (6.15 ± 5.23%), CD8+ non-T_SCM_(12.6 ± 3.48%), and CD4+ non-T_SCM_ (10.2 ± 8.66%) cells (Additional file 1: Figure S1D E F). By allogeneic activating, inhibiting differentiation with TWS119, sorting CFSEdim cells, and expansion with IL-7 and IL-15, our in vitro protocol was able to prepare about 2 × 10^7^ allo-specific CD8+ T_SCM_ cells from 1 × 10^7^ PBLs. The number of the T_SCM_ cells was sufficient to meet the needs of the following studies.

The T_SCM_ preparation strategy used in this study could be translated into a single epitope-specific T_SCM_ preparation. When the E007 co-cultured with allogeneic PBLs, the precursor frequencies were much higher than those of T cell responses to a single allogeneic epitope. To prepare a single epitope-specific T_SCM_ cells in a large number, a modification with a prolonged cytokine expansion was required (Additional file 2: Figure S2).

### The prepared T_SCM_ cells show stem cell and memory T cell characteristics in vitro

**Fig. 2:**
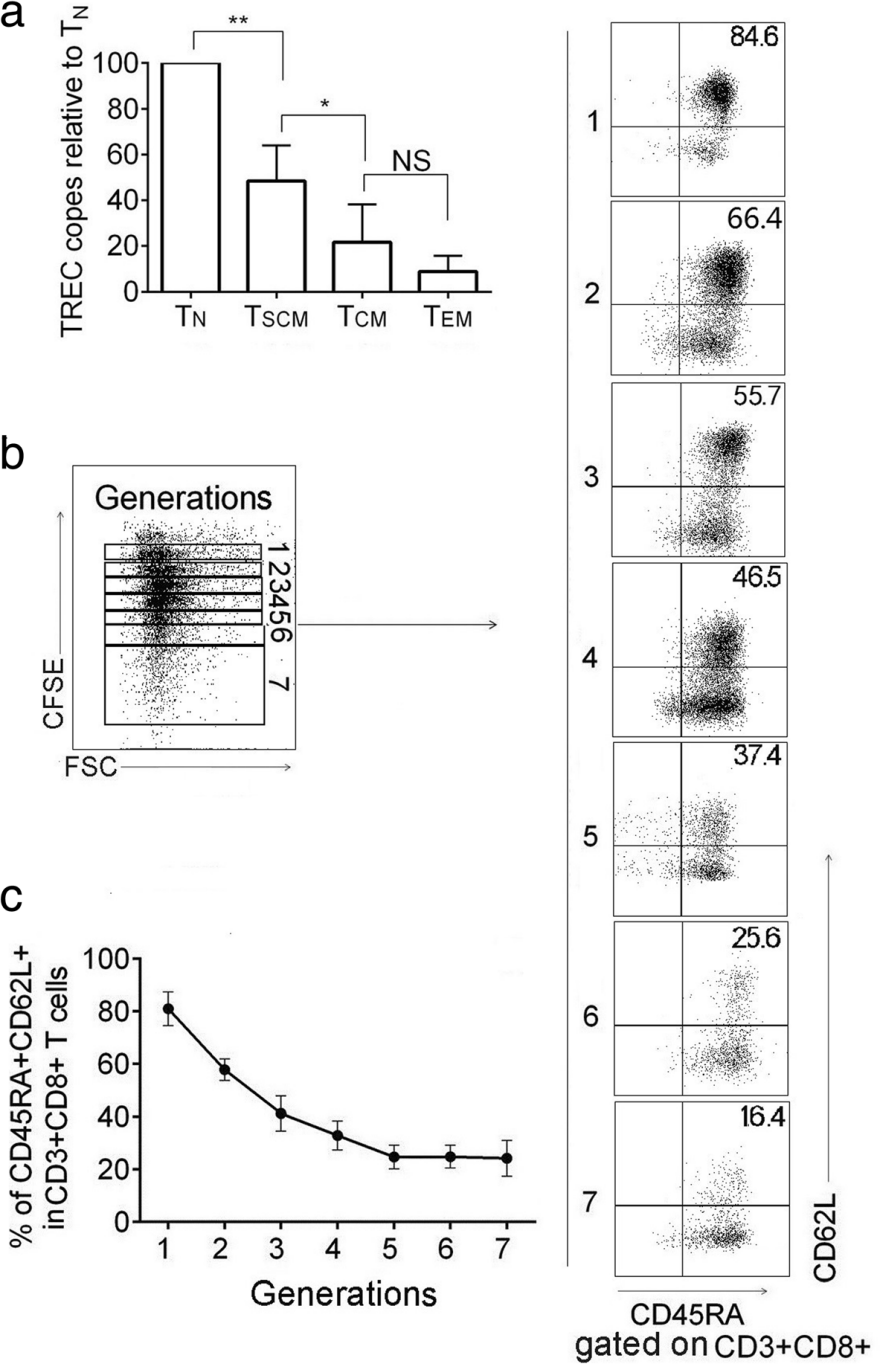
The prepared T_SCM_ cells bear the ability of self-renewing. **a** T_N_, T_SCM_, T_CM_, and T_EM_ cell subsets in CD8+ T cells were sorted by their corresponding phenotypes from the co-culture on day 7. TREC copy number in the sorted subsets relative to T_N_ cells was shown. Data are represented as mean ± SD of four individual experiments. **b** The prepared T_SCM_ cells were relabeled with CFSE and exposed to 200 U/ml of IL-2 for 10 days, the proliferating daughter cells divided into 7 generations (left panel). In each generation, there were a portion of CD8+ T cells expressing CD62L and CD45RA (right panel). Representative FCM plots were shown. **c** The frequency of T_SCM_ (CD45RA+ CD62L+) cells in CD8+ T cells was analyzed at each CFSE dilution peak. Data are represented as mean ± SD of four individual experiments. (*p < 0.05; **p < 0.01, and NS, *p* > 0.05)

To examine the self-renewal capacity, T_N_, T_SCM_, T_CM_, and T_EM_ cells were sorted by their corresponding phenotypes from the co-culture on day 7 (Fig. 1b). The content of T cell receptor rearrangement excision circles (TRECs) in each subset was examined by real-time qPCR. As TRECs cannot be replicated while cell division, the content of TRECs reflects the frequency of T cell proliferation. Results showed that T_N_ had the highest content of TRECs. T_SCM_, T_CM_, and T_EM_ cells possessed less TRECs, confirming they were differentiated from T_N_ through T cell clonal proliferation (Fig. 2a). The content of TRECs in T_SCM_ cells was between those of T_N_ and T_CM_ cells, suggesting T_SCM_ was at the earliest stage after T_N_ activation.

The prepared T_SCM_ cells were relabeled with CFSE and exposed to IL-2 (200 U/ml) for 10 days. As IL-2 is a potential promoter for T cell proliferation and differentiation, seven generations of CFSE-diluted daughter cells developed. A portion of T_SCM_ cells (CD45RA+ CD62L+) were found in each generation, indicating that the prepared T_SCM_ cells were able to maintain a stem cell phenotype during proliferation and differentiation (Fig. 2b, c). Collectively, the prepared T_SCM_ cells showed characteristics of self-renewal.

**Fig. 3:**
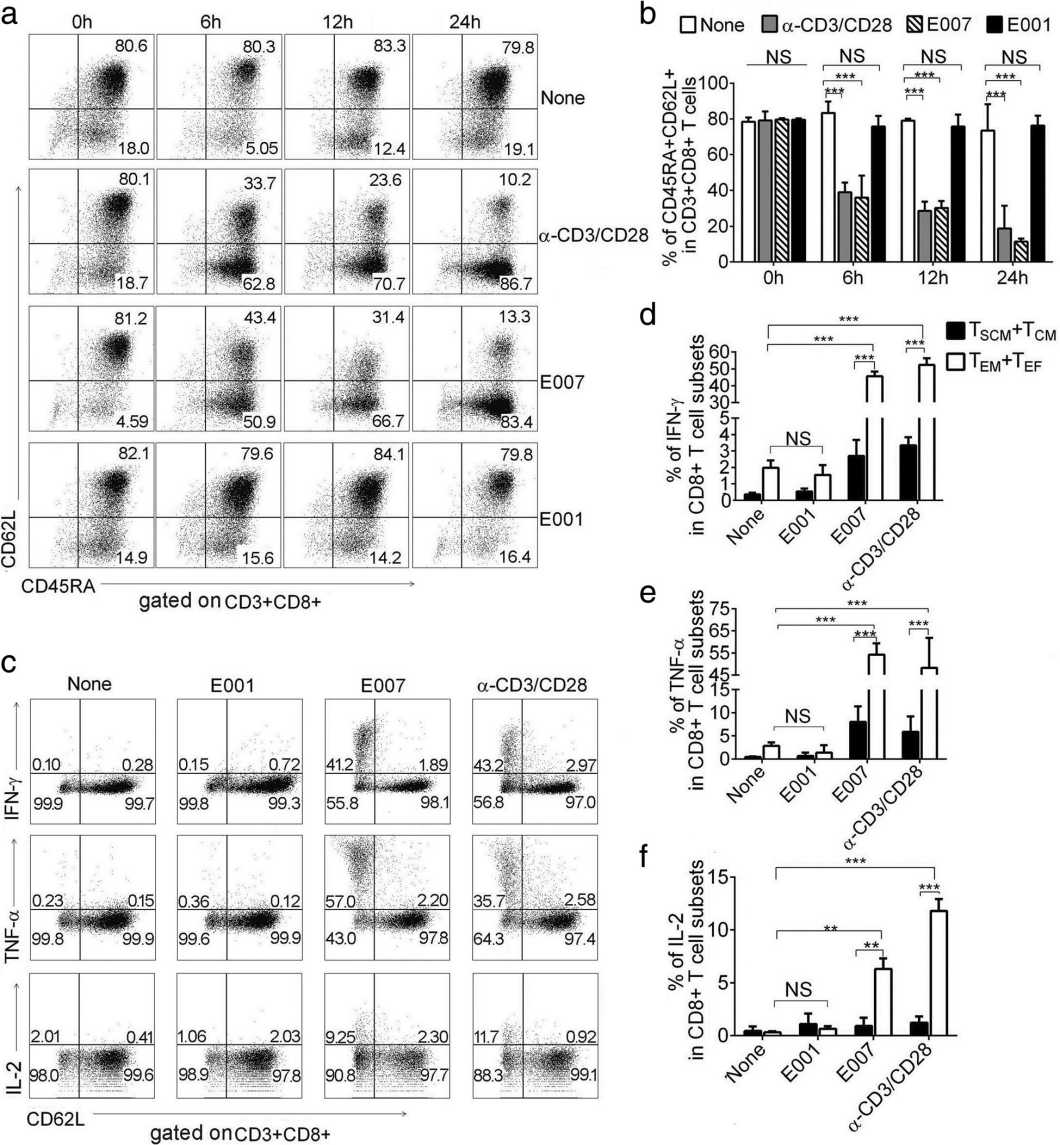
E007 cells stimulate the T_SCM_ differentiate rapidly and the daughter effectors produce cytokines. The prepared T_SCM_ were stimulated with E007, E001, and α-CD3/CD28, respectively. **a**, **b** The T_SCM_ differentiated into effector T cells upon the stimulation with E007. CD3+ CD8+ T cells expressing CD45RA and CD62L (T_SCM_ cells) were detected at indicated hours after stimulation. Representative FCM plots were shown (**a**). Data are represented as mean ± SD of four individual experiments (**b**). **c**–**f** Effector T cells differentiated from the T_SCM_ produced cytokines. Intracellular IL-2, TNF-α, and IFN-γ production of the T cell subsets were detected. Gating by CD62L, the T cells were divided into CD62L+ (T_SCM_ and T_CM_) and CD62L- (T_EM_ and T_EF_) cells, the T_EM_ and T_EF_ subsets showed the cytokine positively stained cells. Representative FCM plots (the percentages indicated the cytokine producing cells in CD62L+ and CD62L- cells, respectively) (**c**) and the frequencies of IFN-γ (**d**), TNF-α (**e**) and IL-2 (**f**) positive cells in corresponding CD8+ T cell subsets were shown. Data are represented as mean ± SD of four individual experiments (**p < 0.01; ***p < 0.001, and NS, *p* > 0.05)

To check memory T cell characteristics, the prepared T_SCM_ cells were incubated with the cognate stimulator E007, HLA class I mismatched E001, and α-CD3/CD28 beads. Phenotypic analysis revealed that the E007-restimulated T_SCM_ cells showed vigorous differentiation in 6 h, which became more intensive with prolonged stimulation time. A similar response was observed in the α-CD3/CD28-stimulated T_SCM_ cells. While the E001-stimulated T_SCM_ cells showed no significant differentiation after 24 h, the response was similar to that of non-stimulated T_SCM_ cells (Fig. 3a, b). In addition, after 24 h of incubation with E007 and α-CD3/CD28, we found that T_SCM_ cells in the incubation showed no significant change in absolute number (Additional file 3: Figure S3E)_,_ suggesting that T_SCM_ cells were able to self-renew during proliferation and differentiation.

CD8+ T cell mediated immune effectors include production of cytokines and killing of target cells. To investigate production of cytokines, the T_SCM_ cells were stimulated with E007, E001, and α-CD3/CD28 beads, then intracellular IL-2, TNF-α, and IFN-γ production was measured. The E007-restimulated T_SCM_ cells showed more positive cells of these cytokines. Gating with CD62L, the incubated bulks were divided into CD62L+ (T_SCM_ and T_CM_) and CD62L- (T_EM_ and T_EF_) cells, most of the cytokine positive cells were T_EM_ and T_EF_ cells (Fig. 3c). Meanwhile, gating with CD45RA, we found that both T_EM_ and T_EF_ cells showed similar frequency of the cytokine positive cells (Additional file 3: Figure S3A-D). Whereas, the T_SCM_ cells in the incubation produced almost background IL-2, and only a low level of TNF-α and IFN-γ. The T_SCM_ cells responded against α-CD3/CD28 in a similar profile of the cytokine production to that against E007. In contrast, the T_SCM_ cells stimulated with E001 showed frequency of cytokine-positive cells in the same way as that with non-stimulation (Fig. 3c–f). Next, we moved to cytotoxicity assay, the T_SCM_ co-cultured with E007 or E001 for 24 h at a ratio 5:1. Killing rate of the T_SCM_ and daughter cells against E007 was higher than that against E001 after 8 h (Additional file 3: Figure S3F, G). These results showed that the prepared T_SCM_ cells were E007-specific and able to differentiate rapidly into effector T cells after stimulated by the same antigen.

### The prepared T_SCM_ cells are able to implant and effectively remove target cells in vivo

**Fig. 4:**
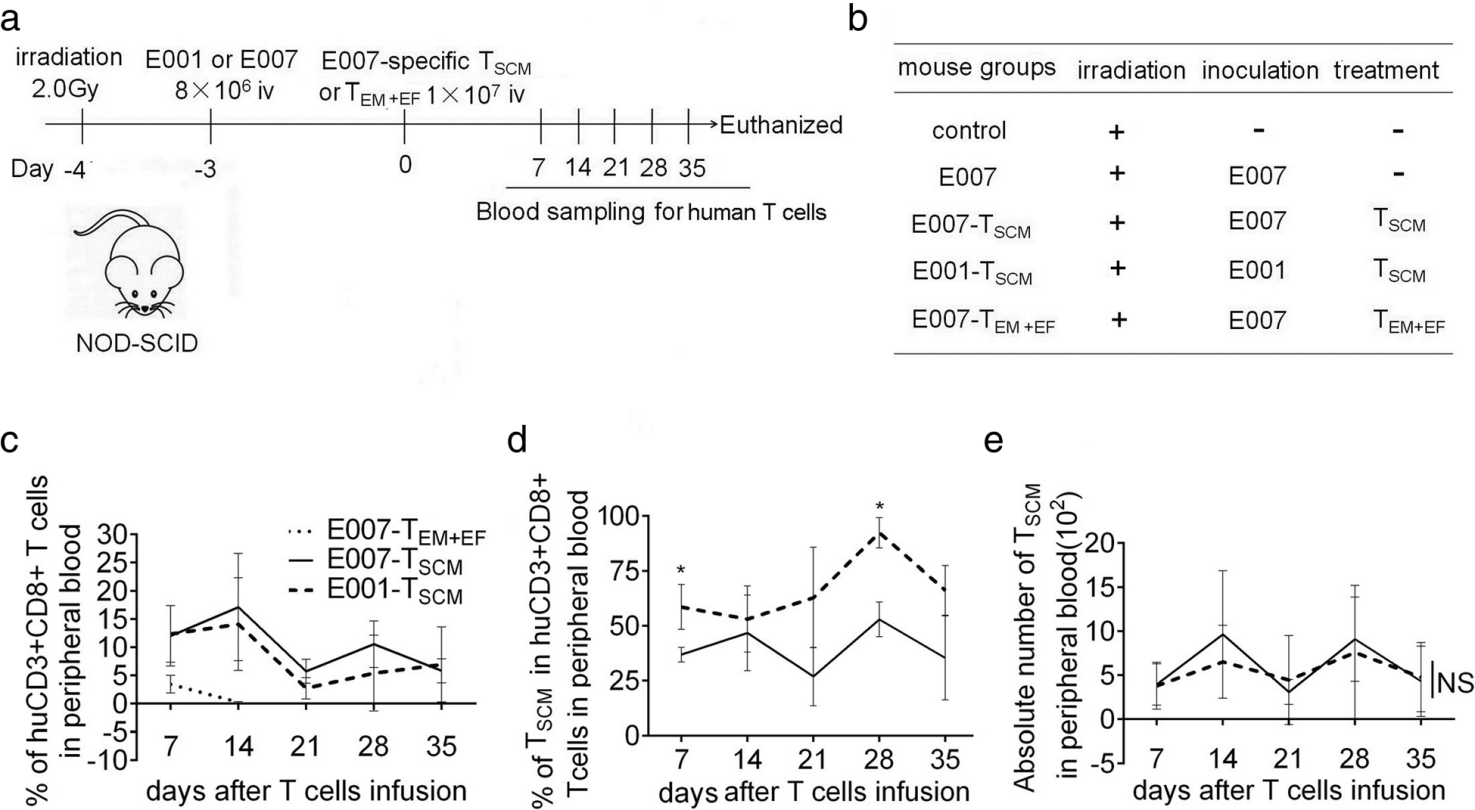
The T_SCM_ are transferred into LCL-burden mice for stem and memory T cell behavior. **a b** Adoptive transfer protocol of the T_SCM_. On day − 3, irradiated mice were inoculated with E007 or E001 cells to set up the LCL-burden mouse model. On day 0, E007-specific T_SCM_ or T_EM_ and T_EF_ (T_EM + EF_) were transferred into the mice intravenously (iv). Blood sampling was taken once a week for 5 weeks after the T cell treatment. On day 35, the mice were sacrificed for detection of human T cells and LCL cells in spleen and bone marrow. **c**–**e** The prepared T_SCM_ cells had superior persistence in vivo. The frequency of human CD3 + CD8+ T cells (**c**), the frequency of T_SCM_ cells (CD45RA+ CD62L+) in human CD3+ CD8+ T cells (**d**), and the absolute number of T_SCM_ cells in 50 μl blood (**e**) were detected by FCM. Data are represented as mean ± SD of five mice. (*p < 0.05; NS, *p* > 0.05)

**Fig. 5:**
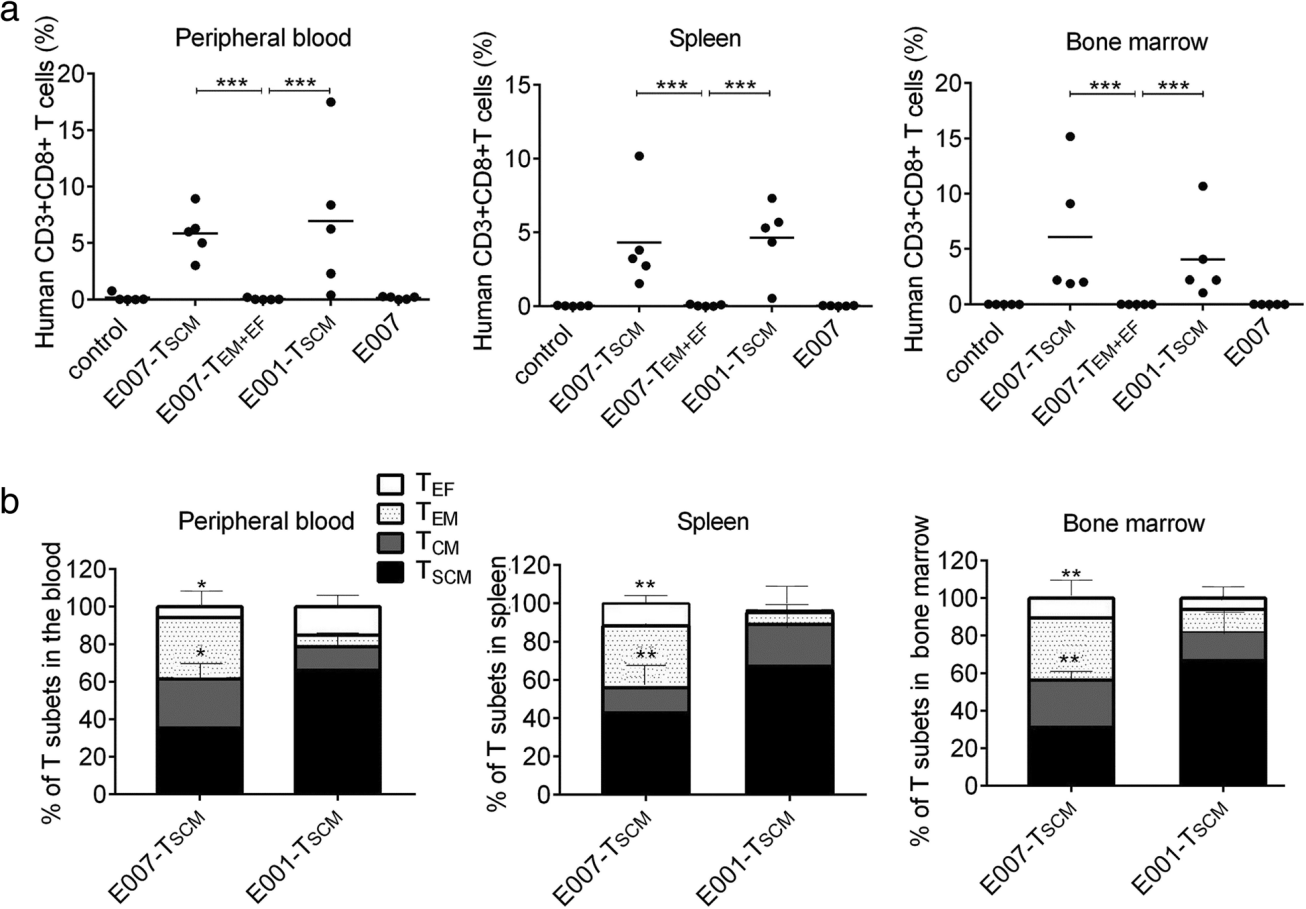
The prepared T_SCM_ cells are able to reconstitute T cell subsets in vivo. The mice were euthanized on day 35. **a** Human CD3 + CD8+ T cells were detected by FCM in peripheral blood, spleen, and bone marrow, respectively. The T_SCM_-treated mice (E007-T_SCM_ and E001-T_SCM_) showed more human CD3 + CD8+ T cells. **b** The distribution of human CD3 + CD8+ T cells subsets (T_SCM_,T_CM_, T_EM_,T_EF_) was detected by FCM in peripheral blood, spleen, and bone marrow, respectively. More effector T cell subsets (T_EM_ and T_EF_) were developed in the E007-T_SCM_ mice. Data are represented as mean ± SD of five mice. (*p < 0.05; **p < 0.01, and ***p < 0.001)

**Fig. 6:**
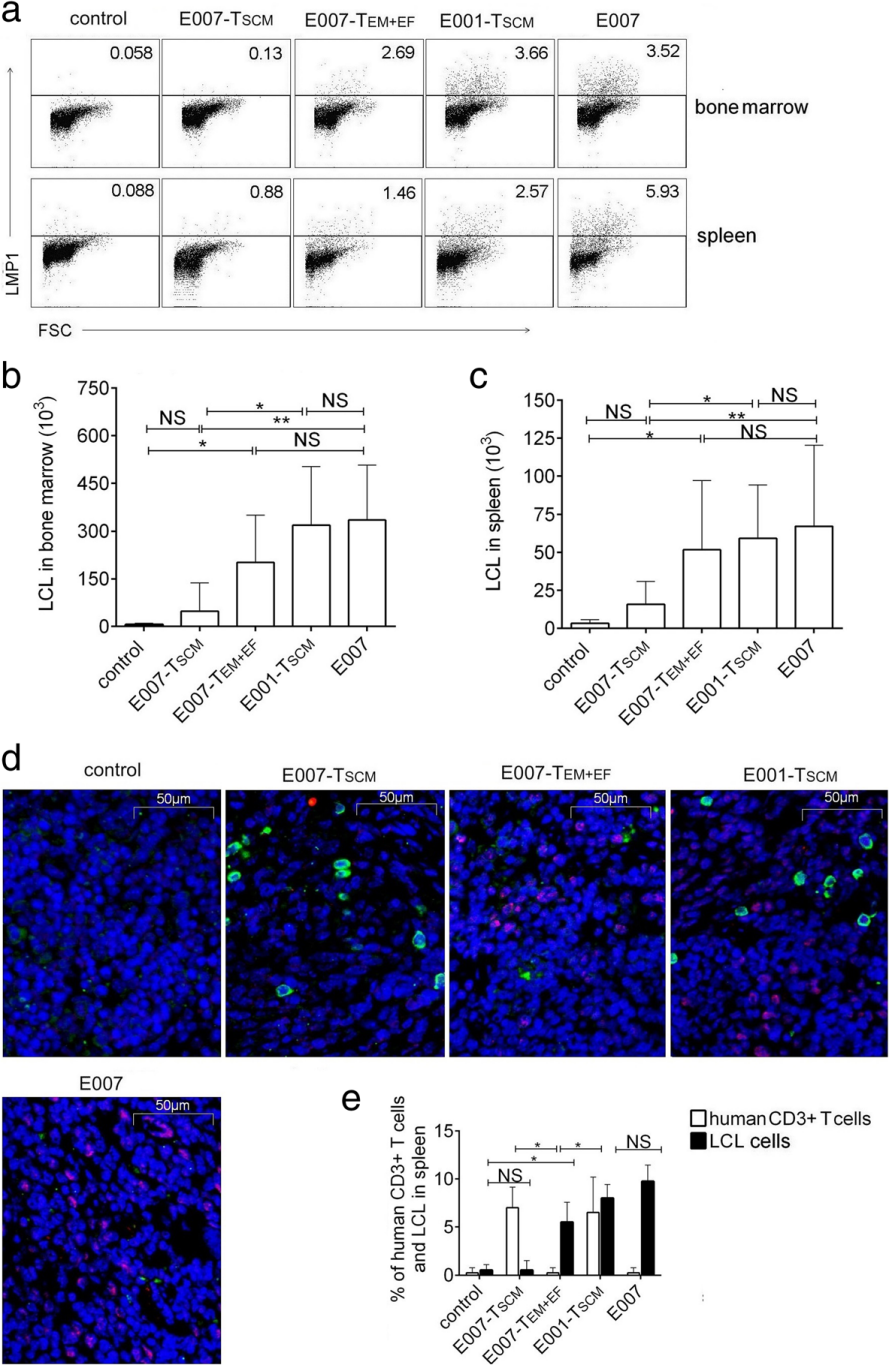
The T_SCM_ cells are able to eradicate allo-antigen-specific targets in vivo. The spleen and bone marrow were detected for the residual LCL cells by intracellular LMP1 staining using FCM. **a**–**c** Representative FCM plots (**a**) and the absolute number of LCLs in bone marrow of two femurs (**b**) and in 100 mg spleen tissue (**c**) were analyzed. **d** Spleen sections were observed for LCLs and human T cells by immunofluorescence. Representative illustrations of immunofluorescent labeling of human CD3 (green), LMP1 (red), and nucleus staining by DAPI (blue) for spleen sections were shown (× 400). **e** Date showed the mean frequencies of human CD3 and LMP-1-positive cells in 4 fields chosen randomly (100 cells each) for each mouse spleen section. The residual LCLs in the E007-T_SCM_ mice were similar to that in the control mice without LCL inoculation, the rest mouse groups showed relatively more LCL cells. Data are represented as mean ± SD of five mice (*p < 0.05; **p < 0.01; ***p < 0.001, and NS, *p* > 0.05)

To evaluate capacity of persistence and rejecting target cells in vivo of the prepared T_SCM_ cells, human LCL cells E001 or E007 were inoculated intravenously into NOD/SCID mice to establish LCL-burden mouse model. After 3 days, the LCL-burden mice were treated with either E007-specific T_SCM_ cells or E007-specific T_EM_ and T_EF_ cells (Fig. 4a, b). On days 7, 14, 21, 28, and 35 after T cell infusion, caudal vein peripheral blood samples were taken to detect the frequency and phenotype of human T cells. We found human CD3+ CD8+ T cells in the peripheral blood of the T_SCM_-treated mice at all sampling times. However, in the T_EM + EF_-treated mice (E007-T_EM + EF_), human T cells were detected in peripheral blood on day 7, but not on day 14 or later (Fig. 4c). In the T_SCM_-treated mice, the frequency of T_SCM_ cells in the E007-burden mice (E007-T_SCM_) were lower than that in the E001-burden mice (E001-T_SCM_), but no difference was found in the absolute number of T_SCM_ cells between E007-burden and E001-burden mice (Fig. 4d, e). On day 35, more frequent human T cells were found in the spleen and bone marrow of the T_SCM_-treated mice revealed by flow cytometry (Fig. 5a), and the immunofluorescent staining of the spleen sections showed the similar results (Fig. 6d, e). These findings indicated that the T_SCM_ cells were able to survive long through self-renewal in the mice, and differentiate into other T cell subsets as stimulated with the specific antigens of E007.

To investigate the immune reconstitution capacity of the T_SCM_ cells, peripheral blood, spleen, and bone marrow of T_SCM_-treated and T_EM + EF_-treated mice were tested for human T cells on day 35. The human T cells in the above specimens consisted of T_SCM_ (CD45RA + CD62L+), T_CM_ (CD45RA-CD62L+), T_EM_ (CD45RA-CD62L-), and T_EF_ (CD45RA+ CD62L-). The distribution of T cell subsets revealed more T_EM_ and T_EF_ cells in the E007-burden mice, whereas more T_SCM_ and T_CM_ cells in the E001-burden mice (Fig. 5b). As T_EM_ and T_EF_ cells are at terminal differentiation stage while T_SCM_ and T_CM_ cells at early differentiation stage, our findings suggested the E007-specific T_SCM_ cells differentiated into the other T cell subsets when they came cross the same antigen in the E007 burden mice.

To analyze killing efficacy of the T_SCM_ cells in vivo, on day 35 of the T cell treatment, the residual LCL cells in mice were detected by intracellular LMP1 staining. The LCL cells were mainly found in the spleen and bone marrow of the LCL-burden mice. In the E007-burden mice, the residual LCL cells in spleen and bone marrow of the T_SCM_-treated mice were similar to that of the control mice without LCL infusion (control). Nevertheless, the T_EM + EF_-treated mice carried a significant amount of the residual LCL cells compared to the control mice, but the amount was lower than the E007-burden mice without T cell treatment (E007) (Fig. 6a–c). More residual LCL cells were also found in the immunofluorescent spleen sections of the T_EM + EF_-treated mice than those of the T_SCM_-treated mice (Fig. 6d, e) These data demonstrated the T_SCM_ cells equipped with superior capacity of killing targets of specific antigens in vivo compared to the T_EM_ and T_EF_ cells.

In contrast to the E007-burden mice, the E001-burden mice bore not only less T_EM_ and T_EF_ cells after the T_SCM_ treatment, but also the residual LCL cells as high as that of the E007-burden mice without T cell treatment (Fig. 6a–c). Results of immunofluorescence staining of spleen sections for human T cells and LCLs showed residual LCL cells in E007-burden mice treated with T_SCM_ were similar to those of the control mice. Although the spleen of the E001-burden mice showed a significant number of human T cells, the residual LCL cells were similar to that of the E007-burden mice without T cell treatment (Fig. 6d, e). By the way, the human T cell-treated mice showed no sign of xenogeneic graft-versus-host disease (xeno-GVHD), such as loss of body weight, back arched, and shed. Collectively, these results indicated that the prepared T_SCM_ cells were able to survive over a long-term in vivo and differentiate into effector T cells to eradicate the target bearing the cognate antigens.

## Discussion

Mature T cells are comprised of cells that are at various stages of differentiation, which are discernible by the expression of surface molecules. T_N_ cells are conventionally defined by the co-expression of the CD45RA, CCR7, and the lymph node homing molecules L-selectin (CD62L) [3]. Similar to T_N_ cells, CD62L and CCR7 are maintained on T_CM_ cells, whereas these molecules are lost on more differentiated T_EM_ cells [3, 35]. T_SCM_ cells, the least differentiated of all distinct memory populations, are identifiable through the expression of markers, including CD62L, CCR7, CD95, and the chemokine (C-X-C motif) receptor 3 [5, 10, 36]. Naïve T cells downregulate CD62L expression after stimulation, as clonal expansion goes on, both CD62L^LOW^ and CD62L^HIGH^ T cell subsets are developed. With real-time tracking of CD8 T-cell divisions in vitro, Kinjyo and co-workers define memory T cells among CD62L^HIGH^ cells. The memory T cells stay CD62L^HIGH^ and proliferate in vitro driven by IL-2, although the division duration is slow, the phenotype and cell cycle duration are inherited by the progeny of the T cells [37]. We identified CD8+ T_SCM_ subset in the co-culture bulks by the phenotypes CD3+ CD8+ CD45RA+ CD62L+ CCR7+ CD95+ CD28+, as our results showed the cells with the phenotypes in the co-culture bulks were of stem cell and memory T cell properties.

A frequently used method to enrich T_SCM_ cells is differentiation inhibition. With antigen priming, antigen-specific CD8+ T_N_ cells proliferate and develop into T_SCM_ cells first and then the other subsets. It is reported that T_SCM_ differentiation can be inhibited by TWS119, which inhibits the GSK-3β and activate Wnt/β-catenin. The inhibition improves the maintenance of ‘stemness’ in mature memory CD8+ T cells which mediated a better anti-tumor response after transferred into mice [15, 38]. Rapamycin is also reported to help the formation of T cell subset at an early stage of differentiation by modulation of mTOR signaling [39–41]. However, rapamycin tended to promote the generation of T_CM_ cells in our study (Additional file 1: Figure S1C), in line with other report [41]. In our co-culture of E007 with allogeneic PBLs, the E007-specific T_SCM_ cells were generated from T_N_ and enriched in the presence of TWS119 which inhibited the T_SCM_ further differentiation. As inhibition by TWS119 results in a T_SCM_ phenotype in human CD8+ T cells [5, 15, 16], our protocol preferred expansion of CD8+ T_SCM_ to that of CD4+ T_SCM_.

The administration of cytokines is another method to expand T_SCM_. Cytokines have important functions related to T cell expansion, differentiation, survival, and homeostasis. Common γ-chain (γc) family cytokines are commonly used in clinical trials, including IL-2, IL-7, IL-15, and IL-21. IL-7 is instrumental for the generation of T_SCM_ cells by binding to IL-7 receptor expressing naive and memory T lymphocytes [42, 43]. Expansion of T_SCM_ required either IL-15 or IL-2; IL-15 proves superior to IL-2 in supporting expansion coupled to preservation of the T_SCM_ phenotype [18, 19]. Activated naive T cells show a higher expression of IL-21 receptor, and IL-21 has been reported to be able to enrich less differentiated T_SCM_ within the total T_SCM_ subset [20, 22, 23, 44]. However, IL-21 exerted few effects on the T_SCM_ expansion in our study. Therefore, IL-7 and IL-15 were used to expand the T_SCM_ cells after the proliferation sorting, resulting in a large number of the T_SCM_ sufficient for the experiments in vitro and in vivo included in our study.

An important feature of stem cells is the self-renewal capacity. Although self-renewal abilities of the prepared T_SCM_ cells were revealed by TREC copy numbers and proliferative history driven by IL-2 in vitro, long-term in vivo implantation is the gold standard for identification of stem cells. Long-lasting survival of human T_SCM_ cells in mice is reported. In serial transplantations model, T_SCM_ cells prove able to persist in secondary recipients suggesting self-renewal abilities [5, 6, 19, 45]. Here, the prepared T_SCM_ migrated to secondary lymphoid organs, such as bone marrow and spleen, showed long-lasting survival potential at least for 35 days after transferred in our model. Importantly, the number of T_SCM_ cells in the blood samples was observed consistent during this study, suggesting the self-renewal capacity of the transferred T_SCM_ cells in vivo. The implantation and long-term existence of the T_SCM_ cells in the host would be expected to mount durable T cell responses after transferring.

Governing antigen specificity is important for T_SCM_ preparation; various strategies are used for this purpose. For example, HCMV-specific T_SCM_ cells are isolated from peripheral blood T_SCM_ cells of HCMV seropositive donors and enriched in vitro by incubation with HCMV antigen [26]. CD19-specific T_SCM_ cells are prepared from peripheral blood T_N_ and T_SCM_ by transduction with a γ-retroviral vector encoding the CD19-chimeric antigen receptor (CAR) [24]. TCR-transgenic mice are also a source of antigen-specific T_SCM_ cells [46]. In our approach, the E007-specific T cells underwent proliferation in the co-culture bulks, making it feasible to be sorted by CFSE dilution. In our pre-experiment, IL-7 or IL-15 was able to make PBLs proliferation without the E007 stimulation. Hence, no cytokine was added to the co-culture before the sorting, to ensure the E007-specificity of the prepared T_SCM_ cells.

Two HLA class I mismatched LCLs, E007 and E001, were used to examine the antigen specificity of the prepared CD8+ T_SCM_ cells. Rapid differentiation into effector T cells and robust effector functions were observed when the prepared T_SCM_ cells were stimulated with the E007, but not with the E001. Although the E007-specificity was suggested by these in vitro findings, it would be more important to observe the behavior of the prepared T_SCM_ cells in vivo. In our LCL burden mouse model, LCL cells could be found in spleen and bone marrow up to 45 days after transfer into the immunodeficiency mice in our preliminary test. The LCL burden mice showed no measurable side effect during our study, such as loss of body weight, back arched, and shed. Interestingly, the prepared T_SCM_ cells exhibited intensive differentiation into other T cell subsets and effective eradication of LCL targets in the E007-burden mice instead of the E001-burden mice. These observations reflected the prepared T_SCM_ cells were E007-specific.

## Conclusions

Functional allo-specific CD8+ T_SCM_ cells were prepared from human PBLs in a procedure of allogeneic co-culture, differentiation inhibition, proliferation sorting, and cytokine expansion. Although our study provided a practical protocol for allo-specific T_SCM_ cell preparation, this method would be adapted to prepare T_SCM_ cells specific for antigen of interesting. As T_SCM_ cells show the implantation and long-term existence in the host after transferring, the preparation of antigen-specific T_SCM_ cells is crucial for T cell adoptive immunotherapy.

## Additional files

Additional file 1: Figure S1. Enrichment of T_SCM_ cells by differentiation inhibitors and the lymphocyte distribution changes in the cultural bulks over the T_SCM_ preparation course. **A**, **B** TWS119 exhibited concentration-dependent enrichment of T_SCM_ in the alloreactive co-culture, 5 μM of TWS119 was the best concentration. T_SCM_ cells in frequency (**A**) and in absolute number (**B**) were shown. **C** Differentiation inhibition by rapamycin was more likely to enrich T_CM_ instead of T_SCM_. **D**–**F** After allogeneic activation, differentiation inhibition, proliferation sorting, and cytokine expansion, lymphocyte distribution in the cultural bulks was revealed by FCM over the T_SCM_ preparation course. CD3 + CD4+ T cells, CD3 + CD8 + T cells and CD3- cells in the cultural bulks were detected, and the proportion of CD3 + CD8+ T cells was increased over time (**D**). The majority of cultural bulks were CD3 + CD8+ T_SCM_ (60.1 ± 11.2%) on day 14 (**E**). The proportion of CD3 + CD8+ T_SCM_ in CD8+ T cells was increased over time, about 80% of CD8+ T cells were T_SCM_ on day 14 (**F**). Data are represented as mean ± SD of four individual experiments. (JPG 170 kb)

Additional file 2: Figure S2. Our T_SCM_ preparation strategy can be used to generate a single epitope-specific T_SCM_ cells. **A** Procedure for preparation of AFP-specific, allogeneic T_SCM_. T2 cells express only empty HLA-A2 allele and no other HLA allele. Alpha fetoprotein (AFP) is a tumor associated antigen of hepatocarcinoma, the hAFP_158–166_ (FMNKFIYEI) is an HLA-A2 restricted peptide. When pulsed with the AFP peptide, T2 cells were able to present the AFP/HLA-A2 complex. The AFP-specific T_SCM_ were raised by co-culturing HLA-A2 negative (HLA-A2-ve) PBLs and the T2 cells pulsed with the AFP peptide (T2/AFP). In a procedure of an allogeneic co-culture, differentiation inhibition, proliferation sorting and cytokine expansion, the AFP/HLA-A2 complex-specific T_SCM_ cells were produced. **B** Co-culture by mixing of 1 × 10^7^ PBLs and 2 × 10^6^ T2/AFP on day 0, a prolonged cytokine expansion was required to generate 1 × 10^6^ AFP-specific T cells. Data are represented as mean ± SD of four individual experiments. **C**–**E** The prepared T_SCM_ cells were AFP-specific. The prepared T_SCM_ cells were incubated with the T2/AFP and T2 cells pulsed with an irrelevant peptide HBcAg_18–27_ (T2/HBC), respectively. After 4 h incubation, the T cell subsets and their intracellular IFN-γ production were detected. Representative FCM plots (**C**). T_SCM_ cells differentiated more when incubated with the T2/AFP (**D**). The daughter cells showed more frequent IFN-γ positive cells when incubated with the T2/AFP (**E**). Data are represented as mean ± SD of four individual experiments (** *p* < 0.01). (JPG 404 kb)

Additional file 3: Figure S3. The prepared T_SCM_ differentiated into effector T cells stimulated by E007. The prepared T_SCM_ were stimulated with E007, E001 and α-CD3/CD28, respectively. The T cell subsets and their intracellular IL-2, TNF-α and IFN-γ production were detected. The T_SCM_ differentiated into effector T cells upon the stimulation with E007 and α-CD3/CD28. **A**–**D** After 4-h stimulation, both T_EM_ and T_EF_ cells exhibited the similar frequency of the cytokine positive cells. Representative FCM plots (**A**). Gating by CD3+ CD8+ CD62L-, the T cells were divided into CD45RA- (T_EM_) and CD45RA+ (T_EF_) cells. The T_EM_ and T_EF_ subsets showed the cytokine positively stained cells of IFN-γ (**B**), TNF-α (**C**) and IL-2 (**D**). **E** After 24-h stimulation, the absolute number of T_SCM_ remained stable during differentiation. **F**, **G** The cytotoxicity of the T_SCM_ and the daughter cells were E007-specific. E007 and E001 labeled with celltrace, co-cultured with the T_SCM_ at ratio 1:5. Dead cells stained by PI dye were detected by FCM. Representative FCM plots (**F**) and the frequencies of dead cells (**G**) were shown. Data are represented as mean ± SD of four individual experiments (NS, *p* > 0.05; **p < 0.01, and ***p < 0.001). (JPG 722 kb)

## Abbreviations

ACT: Adoptive cell therapy
GVHD: Graft versus host disease
HLA: Human leukocyte antigen
LCL: B lymphoblastoid cell lines
LMP1: Latent membrane protein 1
PBL: Peripheral blood lymphocytes
PBMC: Peripheral blood mononuclear cells
pMHC: Peptide major histocompatibility complex
TCR: T cell receptor
TREC: T cell receptor rearrangement excision circle
