# Supplementary figures and images for: Antigen-specific CD8+ memory stem T cells generated from human peripheral blood effectively eradicate allogeneic targets in mice
Source: Stem Cell Res Ther. 2018 Dec 7;9:337. doi: 10.1186/s13287-018-1080-1 (PMC6286512; doi:10.1186/s13287-018-1080-1)

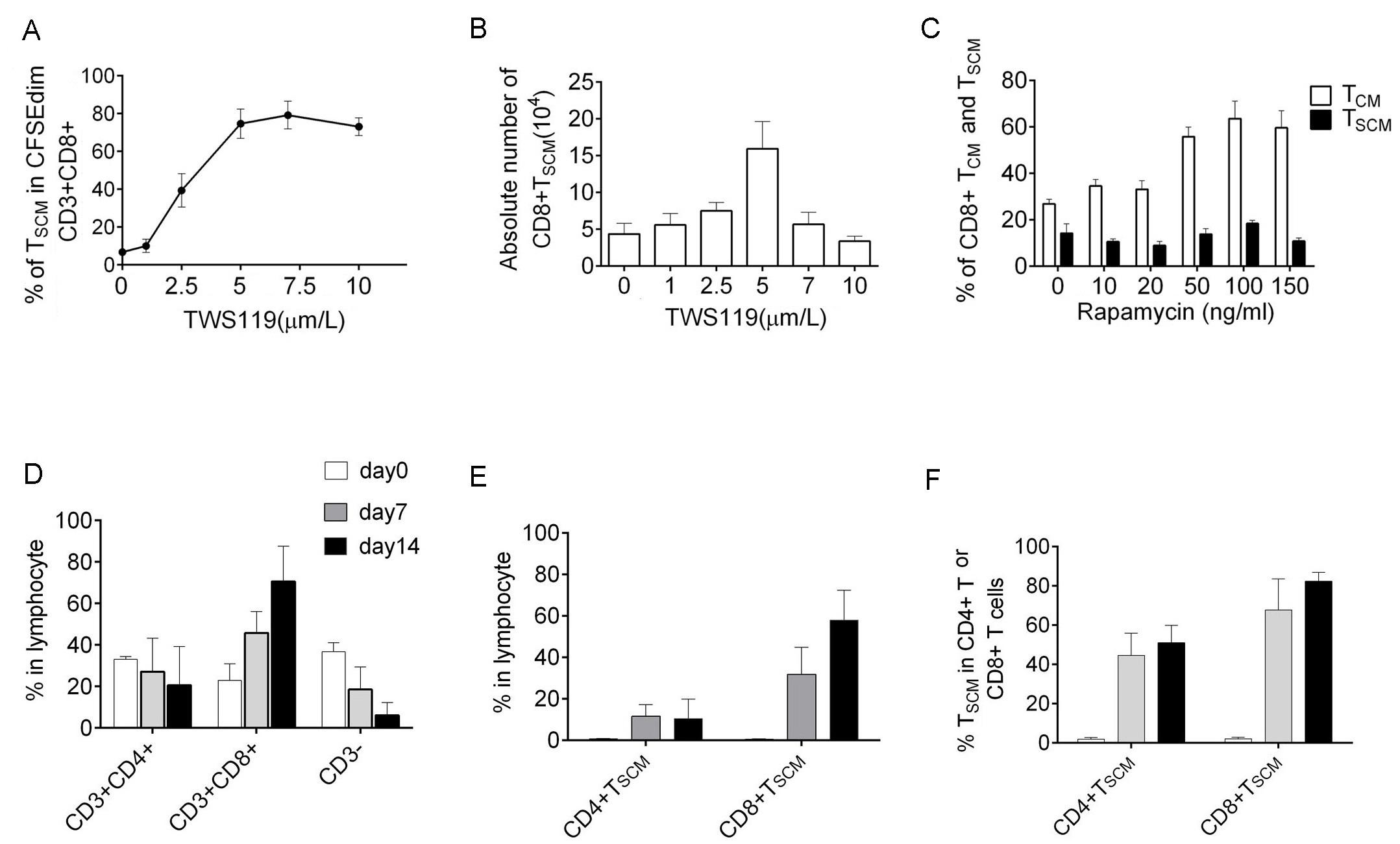

Supplement: Supplementary file 1 — Figure S1. Enrichment of TSCM cells by differentiation inhibitors and the lymphocyte distribution changes in the cultural bulks over the TSCM preparation course. A, B TWS119 exhibited concentration-dependent enrichment of TSCM in the alloreactive co-culture, 5 μM of TWS119 was the best concentration. TSCM cells in frequency (A) and in absolute number (B) were shown. C Differentiation inhibition by rapamycin was more likely to enrich TCM instead of TSCM. D–F After allogeneic activation, differentiation inhibition, proliferation sorting, and cytokine expansion, lymphocyte distribution in the cultural bulks was revealed by FCM over the TSCM preparation course. CD3 + CD4+ T cells, CD3 + CD8 + T cells and CD3- cells in the cultural bulks were detected, and the proportion of CD3 + CD8+ T cells was increased over time (D). The majority of cultural bulks were CD3 + CD8+ TSCM (60.1 ± 11.2%) on day 14 (E). The proportion of CD3 + CD8+ TSCM in CD8+ T cells was increased over time, about 80% of CD8+ T cells were TSCM on day 14 (F). Data are represented as mean ± SD of four individual experiments. (JPG 170 kb) [file 13287_2018_1080_MOESM1_ESM.jpg]

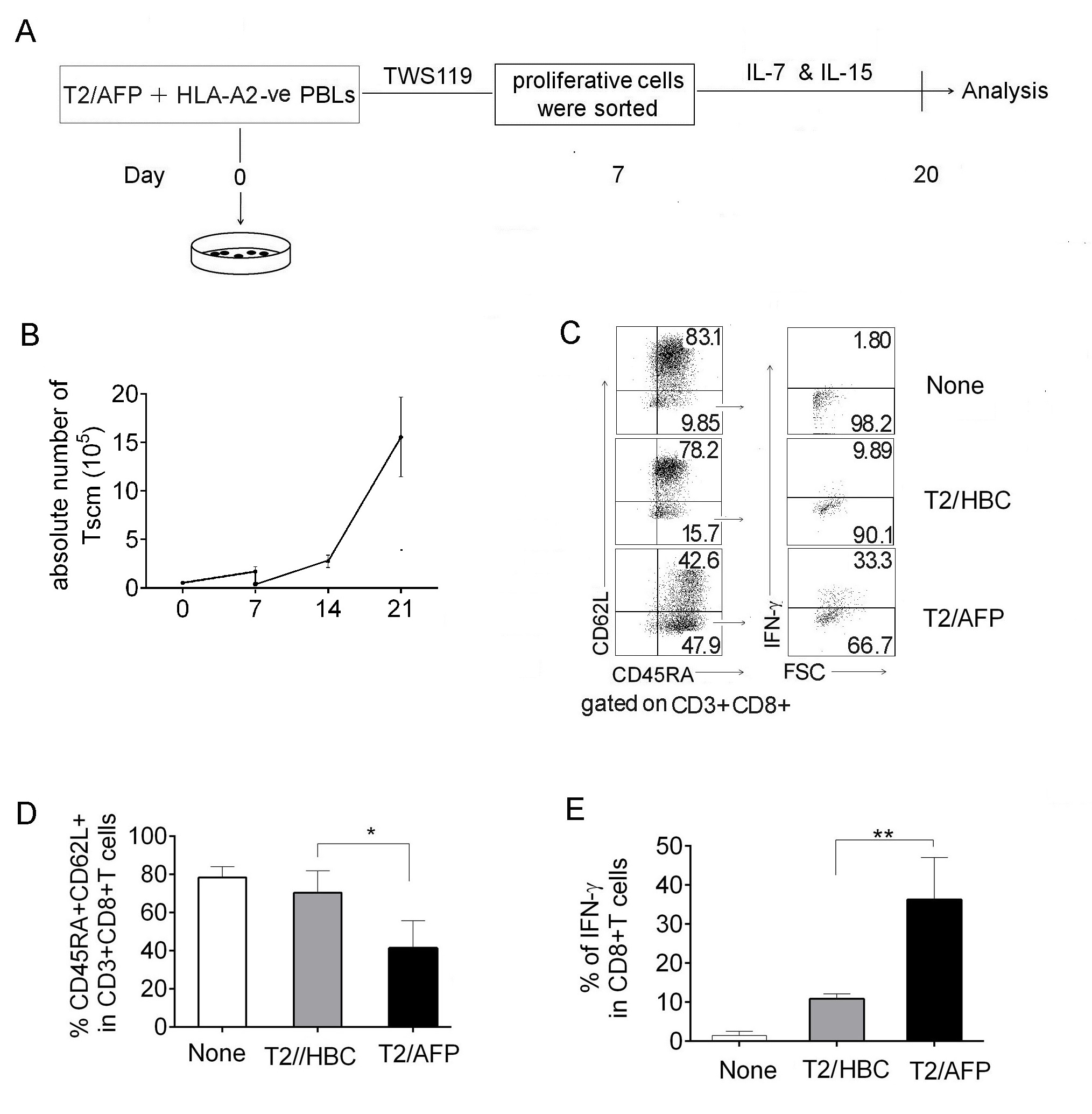

Supplement: Supplementary file 2 — Figure S2. Our TSCM preparation strategy can be used to generate a single epitope-specific TSCM cells. A Procedure for preparation of AFP-specific, allogeneic TSCM. T2 cells express only empty HLA-A2 allele and no other HLA allele. Alpha fetoprotein (AFP) is a tumor associated antigen of hepatocarcinoma, the hAFP158–166 (FMNKFIYEI) is an HLA-A2 restricted peptide. When pulsed with the AFP peptide, T2 cells were able to present the AFP/HLA-A2 complex. The AFP-specific TSCM were raised by co-culturing HLA-A2 negative (HLA-A2-ve) PBLs and the T2 cells pulsed with the AFP peptide (T2/AFP). In a procedure of an allogeneic co-culture, differentiation inhibition, proliferation sorting and cytokine expansion, the AFP/HLA-A2 complex-specific TSCM cells were produced. B Co-culture by mixing of 1 × 10^7^ PBLs and 2 × 10^6^ T2/AFP on day 0, a prolonged cytokine expansion was required to generate 1 × 10^6^ AFP-specific T cells. Data are represented as mean ± SD of four individual experiments. C–E The prepared TSCM cells were AFP-specific. The prepared TSCM cells were incubated with the T2/AFP and T2 cells pulsed with an irrelevant peptide HBcAg18–27 (T2/HBC), respectively. After 4 h incubation, the T cell subsets and their intracellular IFN-γ production were detected. Representative FCM plots (C). TSCM cells differentiated more when incubated with the T2/AFP (D). The daughter cells showed more frequent IFN-γ positive cells when incubated with the T2/AFP (E). Data are represented as mean ± SD of four individual experiments (** p < 0.01). (JPG 404 kb) [file 13287_2018_1080_MOESM2_ESM.jpg]

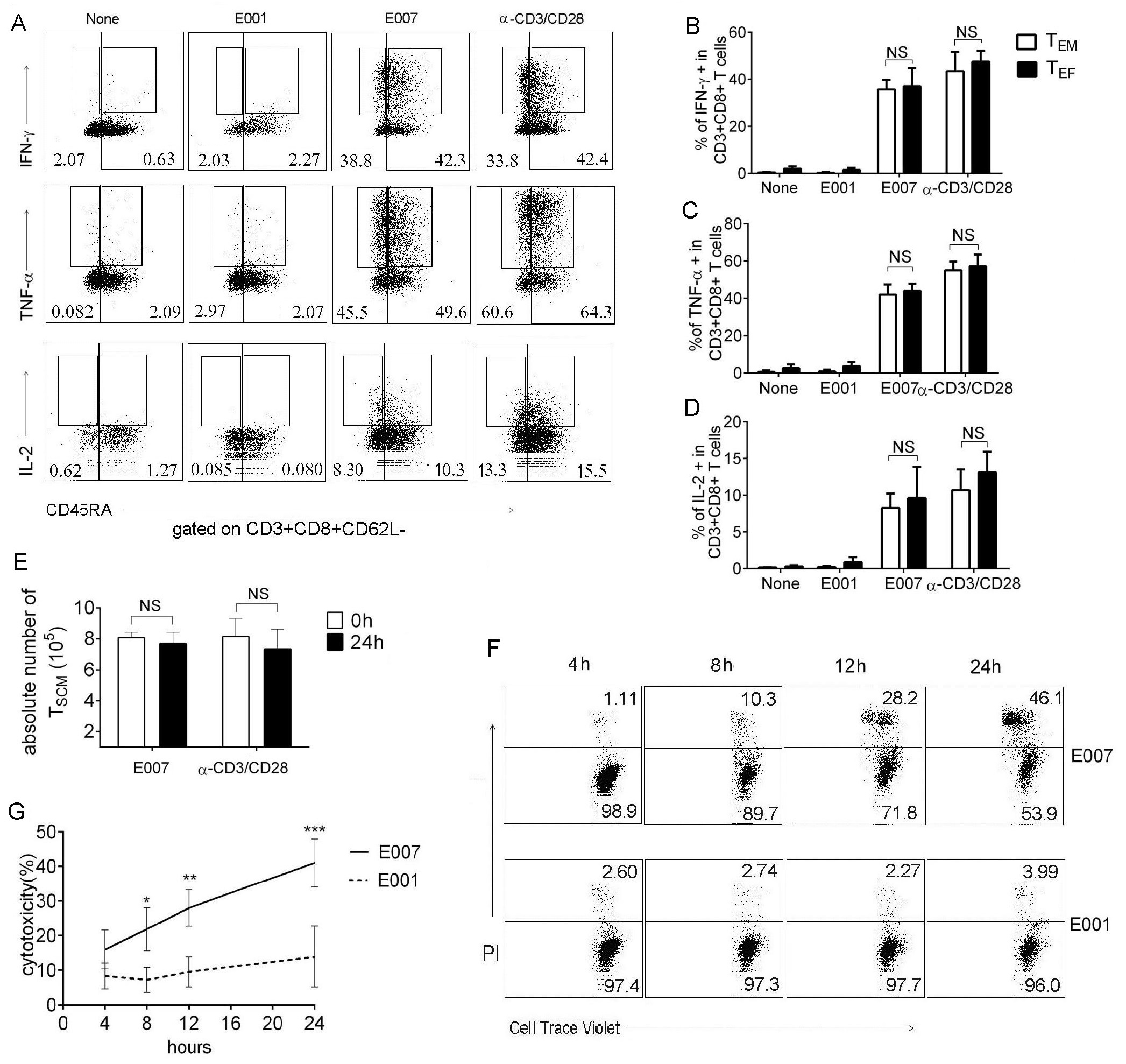

Supplement: Supplementary file 3 — Figure S3. The prepared TSCM differentiated into effector T cells stimulated by E007. The prepared TSCM were stimulated with E007, E001 and α-CD3/CD28, respectively. The T cell subsets and their intracellular IL-2, TNF-α and IFN-γ production were detected. The TSCM differentiated into effector T cells upon the stimulation with E007 and α-CD3/CD28. A–D After 4-h stimulation, both TEM and TEF cells exhibited the similar frequency of the cytokine positive cells. Representative FCM plots (A). Gating by CD3+ CD8+ CD62L-, the T cells were divided into CD45RA- (TEM) and CD45RA+ (TEF) cells. The TEM and TEF subsets showed the cytokine positively stained cells of IFN-γ (B), TNF-α (C) and IL-2 (D). E After 24-h stimulation, the absolute number of TSCM remained stable during differentiation. F, G The cytotoxicity of the TSCM and the daughter cells were E007-specific. E007 and E001 labeled with celltrace, co-cultured with the TSCM at ratio 1:5. Dead cells stained by PI dye were detected by FCM. Representative FCM plots (F) and the frequencies of dead cells (G) were shown. Data are represented as mean ± SD of four individual experiments (NS, p > 0.05; **p < 0.01, and ***p < 0.001). (JPG 722 kb) [file 13287_2018_1080_MOESM3_ESM.jpg]
